# Sun Smart Schools Nevada: Increasing Knowledge Among School Children About Ultraviolet Radiation

**DOI:** 10.5888/pcd14.170202

**Published:** 2017-11-30

**Authors:** Emily Kouzes, Christine Thompson, Cari Herington, Lily Helzer

**Affiliations:** 1 University of Nevada, Reno, School of Community Health Sciences, Reno, Nevada; 2 Nevada Cancer Coalition, www.nevadacancercoalition.org; 3 Nevada Division of Public and Behavioral Health, Carson City, Nevada

## Abstract

**Background:**

Cumulative exposure to ultraviolet radiation (UV) is a risk factor for development of skin cancer. We estimated changes in knowledge, attitudes, and behaviors among Nevada school-age children following implementation of a program to decrease UV exposure.

**Community Context:**

The Nevada Cancer Coalition’s Sun Smart Schools pilot program was implemented in 7 Nevada schools during the 2015–2016 school year. The target population was students at participating schools.

**Methods:**

Participation in the program was voluntary. Students surveyed spanned grades from fourth to tenth. Pre-intervention surveys were conducted at the start of the school year. Post-intervention surveys were conducted at the end of the school year. Changes in knowledge, attitudes, and behaviors were assessed among study participants by using self-reported survey responses.

**Outcomes:**

The Sun Smart Schools pilot program was effective in increasing a broad range of knowledge, attitudes, and behaviors about protection from UV among elementary and middle school students. Students in high school reported an increase in the adoption of selected protective behaviors. However, this population also maintained a positive attitude toward the appearance of tanned skin, indicating susceptibility to competing influences. High school students also did not report any evident change in knowledge about sun protection strategies. Parents reported a decrease in knowledge about UV protection but an increase in adoption of certain protective behaviors.

**Interpretation:**

Our findings are similar to those of previous studies demonstrating that education about the dangers of UV exposure is most effective in younger age groups. Results were mixed in older age groups.

## Background

Exposure to ultraviolet radiation (UV) is a risk factor for the development of skin cancer (1,2). The risk increases with the increasing amount of UV exposure over time (3,4), and exposure during childhood and adolescence can produce harmful long-term effects in adulthood (5), including an increased risk of melanoma. With roughly 300 sunny days per year and an average elevation of 5,500 feet, Nevada residents are at a particularly high risk for the development of skin cancer. Age-adjusted incidence is estimated at 15.6 per 100,000 (6), and age-adjusted deaths at 2.9 per 100,000 per year (7). However, these rates are probably much higher, because incidence of melanoma is underreported in the state (Unpublished master’s thesis, Lensch BT. Going for gold: analyzing death certification only cases in the Nevada Central Cancer Registry to merit registry certification. University of Nevada, Reno, Reno, Nevada; 2016).

Reducing UV exposure at the earliest possible age is essential to reducing skin cancer rates (8–10). Exposure can be reduced or avoided by appropriate use of sunscreen or protective clothing, by seeking shade, and by avoiding indoor tanning (11,12). The Community Guide recommends education and policy interventions in child care centers and primary and middle schools to increase skin cancer prevention behaviors in these vulnerable populations (13).

## Community Context

To decrease the incidence of skin cancer in Nevada, the Nevada Cancer Coalition’s Sun Smart Schools pilot program was conducted during the 2015–2016 school year in 7 schools across Nevada. The goal of this program was to establish healthy sun safety habits among children and teens to prevent skin cancer development later in life by integrating sun safety education with school policy changes designed to support sun safe practices. Sun safe practices are not common in schools, despite their potential to change norms and increase sun safe behaviors that reduce UV exposure at little to no cost (14).

Program participants were students at public and private schools in urban and rural counties and spanned primary school and secondary school age groups. The program was implemented as a partnership between participating schools and the Nevada Cancer Coalition, a statewide nonprofit organization dedicated to cancer control. The program consisted of numerous essential elements but allowed participating schools flexibility in how best to implement these elements. Examples of physical sun-protective elements included asking parents to have their child apply sunscreen before coming to school, providing a 1-ounce bottle of sunscreen to each student for use while on school grounds, and allowing students to wear hats and sunglasses while outdoors during the school day. Teachers were given an age-appropriate curriculum about sun safety to incorporate into their lessons, daily UV index announcements to broadcast over the intercom system, and guest speaker presentations to educate students about UV and sun safety. Each school was required to use an age-appropriate, evidence-based curriculum (the Environmental Protection Agency SunWise for grades kindergarten through eighth, SunSmart U developed by the Skin Cancer Foundation for grades 6 through 12, and CATCH Global Foundation’s Ray and the Sunbeatables [for preschoolers and kindergarten through first grade]). All curricula offered were free, evidence-based, easy to use and teach, and met the schools’ common core requirements.

Schools were also asked to adopt a written policy for sun safety. The Nevada Cancer Coalition provided sample policies and technical assistance to participating schools to help ensure all policies would be effective in reducing UV exposure. The policies required adopting at least one of the following strategies: promotion of sunscreen use, approval of sun protective clothing on campus, and access to shade. We conducted an evaluation of this program via pre-intervention and post-intervention hard-copy surveys of students and parents to measure changes in attitudes and knowledge about sun safety. Parents were not direct participants in the program; however, because parents may be the most important influence in a child’s attitude and behavior about sun safety (15), we theorized that including them to even a limited degree could increase program effectiveness.

## Methods

The Nevada Cancer Coalition used convenience sampling at participating schools to assess pilot program effectiveness. Four distinct subgroups (elementary, middle, and high school students and parents) completed a pre-intervention survey in August or September 2015. A follow-up post-intervention survey was conducted with the same subgroups in May 2016. Each survey was targeted to a fourth-grade reading level and reflected the age-appropriate, evidenced-based curriculum that teachers selected. All surveys were developed in partnership with the Office of Statewide Initiatives at the University of Nevada, Reno, and were validated by that office. A total of 1,441 people completed the pre-intervention survey, and 987 completed the post-intervention surveys for a total of 2,427 total survey responses.

Survey questions were identical at both pre-intervention and post-intervention to assess increased knowledge gain and behavioral and attitudinal changes. The only respondent identifier was the child’s grade, so pre-intervention and post-intervention surveys could not be linked to the same student, and we were able to assess only differences in overall score changes by age group. Responses were analyzed separately for elementary school students (fourth and fifth grade), middle school students (sixth, seventh, and eighth grade), high school students (tenth grade), and parents. Although parents did not receive formal sun-safety education, they received program updates in school newsletters and were encouraged to provide sunscreen and protective clothing for their children while at school.

Because surveys were designed for a reading level of fourth grade and above and were intended to be distributed only to children who met these inclusion criteria, elementary-school–aged students who were younger than this or who did not provide their grade level were excluded from analysis in both the pre-intervention surveys (n = 8) and post-intervention surveys (n = 194). Among middle school students, responses from students who did not provide their grade level were excluded from analysis in both the pre-intervention survey (n = 3) and post-intervention survey (n = 20). Among high school students, because the sun safety curriculum was implemented only in the tenth grade health class, and the pre-intervention survey was completed only by tenth grade students, students in other grades or those who did not provide their grade (n = 25) who took the post-intervention survey were excluded from analysis. A total of 2,177 survey responses were included in the analysis.

Results for the student surveys were weighted by dividing the total number of children enrolled in each grade at each school by the number of responses for that grade, providing a representative response distribution for each grade at each school. Parent surveys were weighted by dividing the total number of students enrolled at each school by the number of parent responses for each school. These weighted responses were used to calculate overall percentages of change among question responses, and χ^2^ testing was conducted to determine if results were significant, meaning the result was not likely to occur randomly and was likely attributable to a specific cause. Additionally, Likert-scale responses were dichotomized in analysis. Survey questions with a Likert-scale response of “most of the time” and “sometimes” were equated with a positive response of “yes,” while those responses of “rarely” and “never” were equated with a negative response of “no.” A 95% confidence level (*P* value < .05) was used to interpret the significance of each result. All analysis was done using SAS version 9.4 (SAS Institute, Inc) software.

## Outcomes

### Elementary school

Overall, elementary school students showed increased belief in the importance of sun safety practices from pre-intervention to post-intervention (Table). Among these students, there was an increase of 8.1 percentage points (*P* = .047) from pre-intervention to post-intervention among those reporting yes to “Do you always or sometimes put sunscreen on when you go outside for a long time?” Additionally, there was an 18.5 percentage-point increase (*P* = .003) in the number students who reported wearing sunglasses post-intervention.

**Table:** Participant Responses to Questions About Sun-Related Attitudes, Behaviors, And Knowledge, From Pre-Intervention To Post-Intervention Surveys, Sun Smart Schools Nevada Pilot Program, 2015–2016^a^

Question and Answer	Elementary School Survey			Middle School Survey			High School Survey			Parent Survey		
	Pre-Intervention, n = 424	Post-Intervention, n = 142	*P* Value^b^	Pre-Intervention, n = 387	Post-Intervention, n = 275	*P* Value^b^	Pre-Intervention, n = 88	Post-Intervention, n = 102	*P* Value^b^	Pre-Intervention, n = 542	Post-Intervention, n = 467	*P* Value^b^
**Sunscreen and tanning behaviors**												
Do you always or sometimes put sunscreen on when you go outside for a long time? (yes)	79.6	87.7	.047	63.4	61.7	.81	49.4	64.3	.04	76.8	79.4	.42
Does your family put sunscreen on when they go outside for a long time? (yes)^c^	81.5	87.6	.16	71.7	69.46	.73	—	—	—	—	—	—
Ever used an indoor tanning bed? (yes)	—	—	—	—	—	—	5.8	8.2	.52	20.2	24.8	.13
**Sun-protective behaviors when outside in the middle of the day**												
Hat used (yes)	42.1	49.2	.24	30.1	49.2	.003	44.8	58.2	.07	48.7	54.2	.14
Sunglasses used (yes)	33.8	52.3	.003	36.9	39.2	.72	46.0	55.1	.22	67.5	71.3	.28
Long-sleeve shirt used when outside in middle of the day (yes)	6.8	8.5	.65	14.9	44.4	<.001	5.8	8.2	.52	14.7	25.6	.005
Seek shade (yes)	51.2	55.0	.54	71.9	65.9	.36	49.4	38.8	.15	57.8	54.6	.41
**Sun-safety and appearance-based attitudes**												
What do you think about protecting yourself from the sun? (important)	87.1	90.7	.37	75.6	72.6	.62	65.5	66.3	.91	89.3	83.1	.03
Do you and your friends look better with a suntan? (yes)	24.0	17.8	.13	19.0	11.9	.03	57.5	60.2	.71	27.7	34.6	.05
Is spending time in the sun healthy? (yes)	18.3	18.4	.76	21.0	20.4	.76	28.7	29.6	.11	14.5	16.9	.03
**Sun-safety knowledge**												
Can you get a sunburn on a cloudy day? (yes)	32.7	42.8	.20	36.5	56.6	.01	86.2	77.6	.14	74.2	68.8	.06
Can spending time in the sun during childhood lead to skin cancer when you are older? (yes)	—	—	—	—	—	—	77.0	81.6	.71	73.9	60.9	<.01
Does a base tan protect your skin from sun damage? (no)	—	—	—	—	—	—	26.4	35.7	.38	51.0	50.9	.85

Abbreviation: —, data not collected for this age group.

a Values are weighted percentages unless otherwise indicated.

b *P* values calculated using χ^2 ^analyses.

c Children at one elementary school received a survey without this question and were thus excluded from the analysis of this question.

The elementary-school–aged students were asked one knowledge-based question reflecting the curriculum provided to their teachers: “Can you get a sunburn on a cloudy day?” There was a 10.1 percentage-point increase in yes responses from pre-intervention to post-intervention, and a 6.8 percentage-point decrease of “I don’t know” responses.

### Middle school

Middle school students showed an overall decrease in attitude toward the importance of sun safety practices from pre-intervention to post-intervention. However, middle school students showed a significant increase of 19.1 percentage points (*P* = .003) among those who reported wearing a hat, and a 29.5 percentage-point increase (*P* < .001) in those who reported wearing a long-sleeve shirt when outside in the middle of the day. Despite some negatively changed behaviors surrounding sun safety, there was a significant 7.1 percentage-point decrease in children reporting that they felt they and their friends looked better with a suntan, with only 11.9% of middle school students reporting they felt this way post intervention.

Middle-school–aged students were asked one knowledge-based question that reflected the curriculum provided to their teachers: “Can you get a sunburn on a cloudy day?” There was a significant increase of 20.1 percentage points in yes responses from pre-intervention to post-intervention, and a decrease of 12.4 percentage points in “I don’t know” responses.

### High school

High school students who participated in the Sun Smart Schools program showed an overall increase in practicing sun safe behaviors with a significant 14.9 percentage-point increase (*P* = .04) in sunscreen use and a nonsignificant increase of 13.3 percentage points (*P* = .07) when outside for a long time from pre-intervention to post-intervention. They also increased their use of hats when outdoors. Despite this, attitudes about the importance of protecting themselves from the sun did not change much from pre-intervention to post-intervention, with a minor increase from 65.5% to 66.3% of respondents saying it was important or very important and most high school students maintaining a positive attitude toward the appearance of tanned skin.

High school students were asked 3 knowledge-based questions reflecting the curriculum provided to their teachers. Responses to the question, “Can you get a sunburn on a cloudy day?” were surprising: 86.2% of pre-intervention respondents answered yes but only 77.6% of respondents answered yes at post-test. The second knowledge-based question asked if they thought spending a lot of time in the sun in childhood could lead to skin cancer when they were older. There was a nonsignificant increase in yes responses of 4.6 percentage points, and a slight decrease in both no and “I don’t know” responses. Lastly, students were asked if they thought a base tan helps protect their skin from sun damage. There was a nonsignificant increase of 9.3 percentage points in correct no responses in the post-intervention survey, and a decrease of 6.7% in “I don’t know” responses.

### Parents 

Although parents were not directly exposed to the program, they received information and updates in newsletters and, potentially, through their children. Even with this limited exposure, there was a significant increase of 10.9 percentage points in parents who reported wearing a long-sleeve shirt when outside in the middle of the day. Responses to 3 knowledge-based questions showed some promising results, but only one, “Do you use a long-sleeve shirt when outside in the middle of the day,” showed a significant increase (*P* = .005). Parents also reported a decrease in the attitude-based question, “What do you think about protecting yourself from the sun”; however, most parents (83%) still found sun protection to be important even at post-survey. Opportunities to expand parent participation could be examined in the future.

### Notable outcome difference among subpopulations

Most elementary-school–aged children felt that protecting themselves from the sun was important in both the pre-intervention survey (87.1%) and post-intervention survey (90.7%). By high school, this dropped to 65.5% pre-intervention and 66.3% post-intervention. In addition, at post-intervention survey only 17.8% of elementary school students felt that they looked better with a tan, whereas 60.2% of high school students felt that they looked better with a tan.

## Interpretation

Behavior changes to reduce UV exposure were seen among all groups. Sunscreen use increased among elementary school and high school students. Middle school students and parents increased their use of long-sleeve shirts when outside in the middle of the day, high school students increased hat use, and elementary school students increased use of sunglasses. The Sun Smart Schools program helped facilitate the availability of sunscreen by asking parents to encourage sunscreen application each morning and to provide sunscreen for use while on the school campus. Additionally, each school was encouraged and supported by the Nevada Cancer Coalition to write new policies supporting the wearing of hats, sunglasses, and other protective clothing when outside, encouraging an atmosphere of sun safety and allowing students to protect themselves.

Students in elementary school were the only group that showed significant knowledge gains pre-intervention to post-intervention. Knowledge deficits among other age groups could be due to poor use of the curriculum provided and may point to the need for additional support from the Nevada Cancer Coalition to participating schools and classrooms. Research highlights the importance of partnerships and program advocates within schools and the community to create robust and lasting sun safety programs, so added support within Nevada’s school system may be needed to encourage full participation (16).

Changing attitudes about sun safety is challenging, especially among high school students. The Centers for Disease Control and Prevention has developed research-based recommendations for interventions to prevent skin cancer (17). Although education and policy approaches in primary school settings (kindergarten through eighth grade) are effective in changing behaviors, insufficient evidence is available to show that educational approaches or activities designed to influence behaviors or attitudes are effective for high school students and parents. The difference in effectiveness between age groups, and the fact that high school students maintain a positive attitude toward the appearance of tanned skin even when acknowledging the risk tanned skin represents, illustrates the conflict high school students may experience in desiring to adhere to social norms despite knowledge of risks. It also highlights a gap in the effectiveness of the existing curriculum to fully address social norm change.

Participation within each school varied by grade level and individual classrooms. Because of this, a true count of total participants reached in the pilot program is not available, and overall response rates to the pre-intervention survey and post-intervention survey cannot be calculated. An additional limitation to this pilot program was that no demographic information was captured for the survey respondents, so differences in participation by sex or race is not available.

Even with a decrease from pre-test to post- test, the majority of parents (83%) felt protecting themselves from the sun to be important. This suggests that as we age into adulthood, sun safety becomes more important. Results at post-intervention survey showed over 35% of parents felt they looked better with a tan, and although that percentage is much higher than that for elementary or middle school students, it is half of what high school students reported, again suggesting adults are more conscientious about skin health than high school students. A limitation to the analysis of the parent surveys was that they captured no identifiable information, and all responses were voluntary. Therefore, it is possible that the group surveyed post-intervention was different from the group surveyed pre-intervention. Additionally, parents were not given specific curricula and were outside the reach of school policy changes, which helps highlight the need for increased communication and participation with parents moving forward.

The pilot program had many limitations. Perhaps the most challenging was the flexibility allowed schools in the implementation of the various program elements. This likely added variability and created challenges in assessing the effectiveness of the program overall. It is also likely that selection bias occurred because of convenience sampling, and all statistics should be interpreted with caution. It must be considered that those who responded to the survey and those who did not respond represent 2 separate populations; thus, we cannot definitively say that results are representative of the general student–parent population. However, convenience sampling was still appropriate given the nature of the pilot study and large sample size to evaluate program effectiveness. Another limitation was the time at which the post-implementation surveys were conducted. This time period was selected to coincide with the end of the school year and was appropriate for assessing the short-term outcomes of knowledge, attitudes, and behaviors. However, a longer follow-up period would be more desirable to measure the program’s long-term effectiveness. Finally, the program began in the fall and was held throughout the winter with the post-implementation survey conducted in late spring. Some of the positive effects measured may be due to attitudinal changes occurring naturally with the season change and not due to the program’s influence.

Our results represent a starting point for developing, expanding, and tailoring the Sun Smart Schools curriculum and focus within a school-based setting. Although the interventions implemented among elementary and middle school students seemed to affect attitude, knowledge, and behavior, high school students and parents showed less behavior change associated with the program. This information indicates areas for improvement within the Sun Smart Schools program. Continuing to make policy changes within schools to improve accessibility of sun safety practices could influence better sun-protective behaviors from high school students while at school. Additionally, encouraging environmental changes within schools, such as adding shade structures or trees to the campus, are important. Clark County, which houses Nevada’s largest student population and experiences extreme heat, created an initiative to add shade structures to all playgrounds at elementary schools in their district (18), and similar actions should be encouraged in schools across Nevada. As the program goes forward, we will collect information on sex and race to find gaps in program elements and to address them as needed. One goal of this program is to provide long-term results to help bridge the behavioral and attitude gap between elementary school age and adulthood by building a solid foundation of knowledge and behaviors that change the way adolescents act as they age. By exposing children to a sun safety curriculum and by providing protection at school throughout primary and secondary education, we hope to create a culture of sun safety and thus reduce the incidence of skin cancer in Nevada.
